# A novel circulating osteoimmunological signature for diagnosis: integrating CXCL2, FYN, galectin-3, and STING in postmenopausal osteoporosis

**DOI:** 10.3389/fphys.2026.1791427

**Published:** 2026-03-24

**Authors:** Shuang Ma, Qi Yao, Xiaoxue Bao, Yukun Li

**Affiliations:** 1 Department of Endocrinology, Hebei Medical University Third Hospital, Shijiazhuang, Hebei, China; 2 Department of Endocrinology, Baoding NO.1 Central Hospital, Baoding, Hebei, China

**Keywords:** CXCL-2, Fyn, galectin-3, osteoimmunology, postmenopausal osteoporosis, STING

## Abstract

**Objective:**

The emerging field of bone immunology has clarified the intricate interactions between the immune system and bone metabolism. This investigation was designed to examine changes in concentrations of critical immune cytokines—C-X-C motif chemokine ligand 2 (CXCL-2), FYN (a Src family nonreceptor tyrosine kinase), Galectin-3 (a β-galactoside-specific lectin), and stimulator of interferon genes (STING)—in postmenopausal osteoporotic women, and to explore their potential as biomarkers for early diagnosis of postmenopausal osteoporosis (PMOP).

**Methods:**

Between June and September 2025, researchers recruited 100 postmenopausal women diagnosed with osteoporosis and 100 with osteopenia from the Third Hospital of Hebei Medical University, who were subsequently allocated to the Osteoporosis and Osteopenia groups, respectively. Concentrations of CXCL-2, FYN, Galectin-3, and STING were ascertained employing enzyme-linked immunosorbent assay (ELISA) across all study groups.

**Results:**

Within the Osteoporosis group, CXCL-2 and FYN concentrations demonstrated marked elevation relative to the NC group, whereas Galectin-3 and STING concentrations showed marked reduction (P < 0.05). Pearson correlation and multiple linear regression analyses revealed that CXCL-2, FYN, Galectin-3, and STING levels were strongly associated with BMD. ROC analysis demonstrated that Galectin-3 exhibited the greatest diagnostic precision for PMOP, with an area under the curve (AUC) of 0.881. Additionally, the combined diagnostic performance of all four factors surpassed that of any single marker.

**Conclusion:**

CXCL-2, FYN, Galectin-3, and STING, as immune-related molecules, are integral to bone immune regulation and are closely associated with the pathogenesis of PMOP, positioning them as potential biomarkers for its early diagnosis.

## 1 Introduction

Osteoporosis, a prevalent condition linked to aging, is distinguished by reduced bone density, impaired bone microarchitecture, enhanced bone fragility, and a markedly elevated susceptibility to fractures (Jehpsson et al., 2021; Rempe et al., 2022). As the global population continues to age, osteoporosis is expected to increasingly burden healthcare systems worldwide, including in China (Bragonzoni et al., 2023; Fan et al., 2023). A hallmark of osteoporosis is its higher incidence in women compared to men, with postmenopausal osteoporosis (PMOP) accounting for the majority of cases among females (Geng et al., 2024). PMOP is a common and significant form of osteoporosis, with its development influenced by several factors, including a marked decline in estrogen levels after menopause, the aging process, and genetic predisposition (Cortet et al., 2024). Recently, bone immunology has emerged as a critical and rapidly evolving research area. Estrogen deficiency induces a chronic, low-grade pro-inflammatory state in which various immune cells and the cytokines they produce affect osteoblast and osteoclast functions *via* pathways such as RANK/RANKL/OPG, disrupting bone homeostasis (Okamoto, 2024). C-X-C motif chemokine ligand 2 (CXCL-2), alternatively referred to as macrophage inflammatory protein 2 (MIP-2), represents a member of the CXC family that participates in various immune and inflammatory processes (Yang et al., 2019). Animal studies have shown that CXCL-2 inhibits osteoblast differentiation while promoting osteoclastogenesis by inhibiting the ERK1/2 signaling pathway (La Cognata et al., 2023). FYN, a nonreceptor tyrosine kinase from the Src family, is crucial in various cellular signaling pathways, including proliferation, differentiation, and survival, and acts as a key mediator between metabolic and immune processes (Xu et al., 2024). FYN might affect the adipogenic differentiation of bone marrow mesenchymal stem cells through the regulation of lipid metabolism (Chen et al., 2023). Galectin-3, a β-galactoside-specific lectin from the protein family, alternatively designated as IgE-binding protein MAC2, L-29, and CPB-35, demonstrates extensive expression throughout multiple tissues. This protein participates in diverse physiological and pathological mechanisms, encompassing inflammation, cell differentiation, fibrosis, and transcriptional regulation (Chen et al., 2020). In the skeletal system, its expression is regulated by Runx2 (Stock et al., 2003). Iacobini et al. demonstrated that Galectin-3 serves as a marker for chondrocyte and osteoblast cell lines, suggesting its involvement in endochondral ossification (Iacobini et al., 2018). The stimulator of interferon genes (STING), which functions as an endoplasmic reticulum transmembrane protein, identifies nucleic acids or cellular membrane constituents derived from pathogenic microorganisms. Serving as a primary defense mechanism against pathogen infiltration, STING performs an essential function in immune modulation. Following cGAMP binding, STING experiences structural modifications that trigger downstream signaling cascades, resulting in the generation of immune mediators including interferons (Gao et al., 2023; MacLauchlan et al., 2023; Mao et al., 2024). Nuclear factor kappa B (NF-κB), a downstream effector, has been shown to mediate the involvement of this pathway in the progression of bone metabolic disorders (Sun et al., 2019; Li C. et al., 2024).

The selection of CXCL-2, FYN, Galectin-3, and STING in this study is hypothesis-driven, based on the concept of osteoimmune interactions in PMOP, aiming to capture both the pro-osteoclastogenic/pro-inflammatory axis (CXCL-2, FYN) and the immunomodulatory/anti-osteoclastogenic axis (Galectin-3, STING). Prior evidence supports the involvement of each factor in bone metabolism: CXCL-2 promotes osteoclastogenesis and is upregulated in bone loss conditions (La Cognata et al., 2023); FYN, a key Src-family kinase, is essential for osteoclast activation and bone resorption signaling (Chen et al., 2023; Xu et al., 2024); Galectin-3 regulates osteoblast differentiation and osteoclast apoptosis, and is implicated in bone inflammatory diseases (Stock et al., 2003; Iacobini et al., 2018; Chen et al., 2020); STING is a novel regulator of skeletal homeostasis, with MacLauchlan et al. (2023). demonstrating its context-dependent role in osteoclastogenesis, where myeloid-specific STING deletion in ovariectomized mice primarily affects trabecular bone volume. While these factors have been individually associated with bone metabolism, their combined investigation as a network in PMOP has not been previously reported.

Despite these insights, the precise roles and mechanisms of CXCL-2, FYN, Galectin-3, and STING in PMOP remain poorly understood. This study, therefore, focuses on evaluating the levels of CXCL-2, FYN, Galectin-3, and STING in patients with PMOP, with the aim of assessing their potential as a combined biomarker panel for early detection of the disorder and exploring the clinical evidence for an osteoimmune network in PMOP pathogenesis.

## 2 Materials and methods

### 2.1 Participant selection

From July to November 2025, 100 postmenopausal women diagnosed with osteoporosis at the Third Hospital of Hebei Medical University were assigned to the Osteoporosis group. The Osteopenia group comprised an additional 100 postmenopausal women diagnosed with osteopenia, whereas 100 postmenopausal women exhibiting normal bone density constituted the normal control group (NC group) for comparative analysis. The inclusion criteria encompassed the following: 1) Participants ranged from 45 to 55 years of age, experiencing natural menopause 5 to 10 years previously; 2) Osteoporosis classification in postmenopausal women relied on T-scores, following World Health Organization guidelines: T-scores ≤ −2.5 standard deviations (SD) signified osteoporosis, T-scores ranging between −2.5 and −1.0 SD denoted osteopenia, and T-scores ≥ −1.0 SD indicated normal bone mineral density (BMD); 3) None of the study subjects had received prior anti-osteoporosis treatment. Exclusion criteria encompassed thyroid or parathyroid disorders, diabetes mellitus, severe cardiovascular diseases, hepatic insufficiency, renal dysfunction, metabolic or genetic bone disorders, malignant tumors, or a history of extended hormone therapy.

### 2.2 BMD detection

BMD measurements at the femoral neck, total hip, and lumbar spine (L1-4) were procured through dual-energy X-ray absorptiometry (DXA). The acquired BMD values underwent conversion to T-scores for diagnostic applications. The T-score was computed by subtracting the measured BMD value from the mean peak BMD of young adults of the same race and gender, then dividing the result by the standard deviation of peak BMD for the same group.

### 2.3 General data collection

Data regarding participants’ age, height, weight, and body mass index (BMI) were documented. BMI was computed utilizing the formula: BMI = weight/height^2^ (kg/m^2^).

### 2.4 Biochemical parameters assays

Liver function assessment involved determining alanine aminotransferase (ALT) and aspartate aminotransferase (AST) levels through substrate enzyme methodology. Serum creatinine (SCr) values were quantified using the sarcosine oxidase approach. Total cholesterol (TC) and triglyceride (TG) measurements were obtained via oxidase-based analysis. Fasting blood glucose (FBG) concentrations were established utilizing the glucose oxidase technique. Calcium (Ca) levels were examined employing the arsenazo III method, while phosphorus (P) levels were quantified via the phosphomolybdic acid technique. Alkaline phosphatase (ALP) levels were analyzed using the AMP buffer method. Parathyroid hormone (PTH), 25-hydroxyvitamin D [25-(OH) D], and calcitonin (CT) levels were determined via chemiluminescence immunoassays. Serum type I collagen cross-linked C-terminal peptide (β-CTX) and serum type I procollagen N-terminal propeptide (PINP) were quantified by electrochemiluminescence immunoassays.

### 2.5 Collection and detection of immune cytokines

Participants contributed 5 mL of fasting peripheral venous blood for laboratory analysis. The specimens were permitted to coagulate at ambient temperature for 30 minutes, subsequently undergoing centrifugation at 3300 revolutions per minute (rpm) for 10 minutes. The resulting supernatants were preserved at −80 °C for additional testing. Serum concentrations of CXCL-2, FYN, Galectin-3, and STING were quantified using ELISA kits (Shanghai Animalunion Biotechnology Co., Ltd.; brand: Animalunion). Hemolyzed samples were excluded from analysis. According to the manufacturer’s guidelines, the measurable ranges for CXCL-2, FYN, Galectin-3, and STING were 15.6 to 1000 pg/mL, 0.156 to 10 pg/mL, 156 to 10,000 pg/mL, and 62.4 to 4000 pg/mL, respectively. All procedures were performed in strict accordance with the kit protocol.

### 2.6 Statistical methods

Statistical analysis was conducted utilizing SPSS 27.0 software. Continuous data following normal distribution are expressed as mean ± SD. The Levene test was employed to assess variance homogeneity. One-way analysis of variance (ANOVA) was utilized for intergroup comparisons, alongside Pearson correlation analysis. Multiple linear regression analysis was implemented to investigate the associations between CXCL-2, FYN, Galectin-3, STING, and relevant factors. The diagnostic performance of CXCL-2, FYN, Galectin-3, and STING for PMOP was assessed through receiver operating characteristic (ROC) curve analysis. Statistical significance was defined as a p-value below 0.05.

## 3 Results

### 3.1 Comparison of baseline characteristics across the three participant groups

No significant differences existed regarding age, FBG, TC, TG, ALT, AST, Ca, P, SCr, or CT levels across the groups (all P > 0.05, as indicated by *post-hoc* analyses in **Table 1**). Nevertheless, BMI demonstrated markedly elevated values in the Osteopenia group relative to the NC group (P = 0.031). The NC group exhibited the highest 25-(OH)D concentrations, with significant differences between all groups (P < 0.001 for all pairwise comparisons). Additionally, PTH concentrations showed marked elevation in both the Osteopenia and Osteoporosis groups when compared to the NC group (P < 0.001 and P = 0.001, respectively). Comprehensive data are depicted in **Table 1**.

**Table 1:** Comparison of general information between the three groups.

	NC group	Osteopenia group	Osteoporosis group
Variables	(n=100)	(n=100)	(n=100)
BMI (kg/m^2^)	24.51 ± 2.12	25.35 ± 2.43^*^	24.76 ± 3.23
Age (years)	49.81 ± 2.27	49.88 ± 2.25	49.82 ± 2.29
FBG (mmol/L)	5.15 ± 0.28	5.21 ± 0.45	5.16 ± 0.26
TC (mmol/L)	4.50 ± 0.31	4.50 ± 0.30	4.50 ± 0.34
TG (mmol/L)	1.22 ± 0.15	1.24 ± 0.15	1.25 ± 0.16
ALT (U/L)	20.06 ± 3.58	20.07 ± 3.02	20.07 ± 3.33
AST (U/L)	19.79 ± 3.31	19.10 ± 2.81	19.62 ± 3.31
SCr (umol/L)	56.66 ± 5.88	57.42 ± 9.08	56.89 ± 6.93
Ca (mmol/L)	2.26 ± 0.62	2.27 ± 1.00	2.25 ± 0.98
P (mmol/L)	1.23 ± 0.14	1.22 ± 0.67	1.20 ± 0.82
25-(OH) D (ng/ml)	32.79 ± 4.83	27.66 ± 3.62^*^	22.73 ± 4.70*^△^
PTH (pg/ml)	34.34 ± 2.93	38.14 ± 8.41^*^	37.47 ± 8.17^*^
CT (pg/ml)	3.24 ± 0.57	3.22 ± 0.67	3.20 ± 0.67

Data were presented as mean ± SD. p values from one-way ANOVA test.

BMI, body mass index; ALT, alanine transaminase; AST, aspartate aminotransferase; Scr, serum creatinine; TC, total cholesterol; TG, triacylglycerol; FBG, fasting blood glucose; PTH, parathyroid hormone; CT, calcitonin.

NC group, normal control subjects; ^*^P<0.05 compared with group NC, ^△^P<0.05 compared with group osteopenia.

### 3.2 Comparison of bone mineral density at different sites among the three groups of subjects

BMD measurements at the left femoral neck, left total hip, and lumbar spine (L1-4) demonstrated markedy diminished values in both the Osteoporosis and Osteopenia groups versus the NC group (all P < 0.001). Detailed information is presented in **Table 2**.

**Table 2:** Comparison of bone mineral density between the three groups.

	NC group	Osteopenia group	Osteoporosis group
Variables	(n=100)	(n=100)	(n=100)
FN BMD(g/cm^2^)	0.89 ± 0.06	0.72 ± 0.05^*^	0.51 ± 0.06*^△^
TH BMD(g/cm^2^)	0.96 ± 0.05	0.86 ± 0.05^*^	0.65 ± 0.09*^△^
LS BMD(g/cm^2^)	1.07 ± 0.06	0.93 ± 0.06^*^	0.72 ± 0.10*^△^

Data were presented as mean ± SD. p values from one-way ANOVA test.

NC group, normal control subjects; *P<0.05 compared with group NC, ^△^P<0.05 compared with group osteopenia.

Abbreviations: BMD, bone mineral density; FN BMD, BMD(Left Femoral Neck); TH BMD, BMD (Left Total Hip); LS BMD, BMD (Lumbar Spine).

### 3.3 Comparison of CXCL-2, FYN, Galectin-3, STING, and bone metabolism markers among the three groups

**Figure 1:**
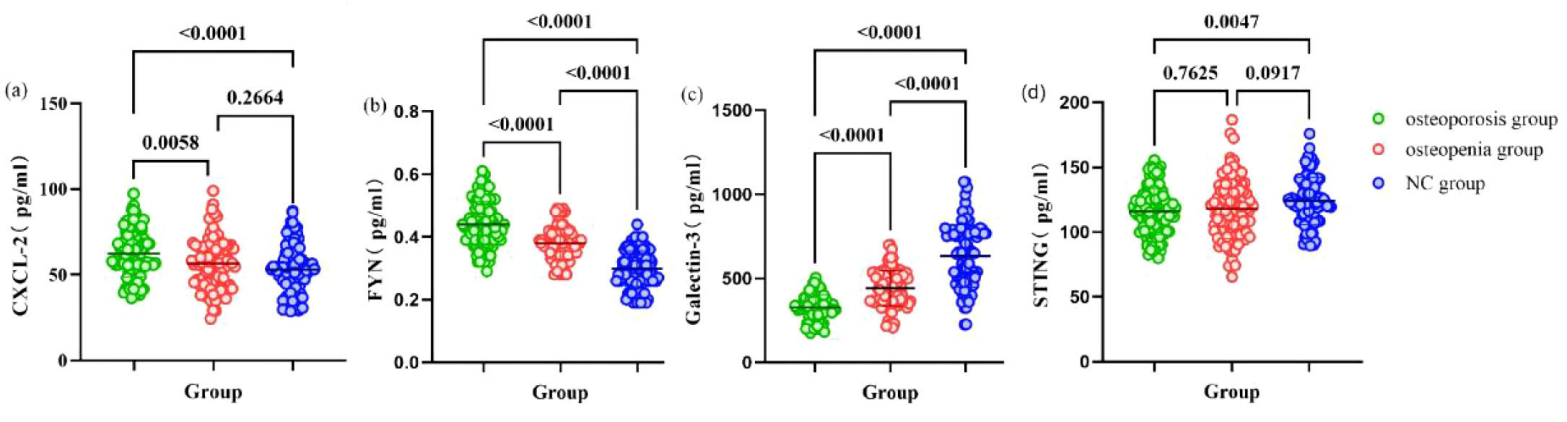
Comparison of CXCL-2, FYN, Galectin-3 and STING levels in patients among the three groups was performed.

As shown in **Table 3** and **Figure 1**, CXCL-2 and FYN concentrations were significantly elevated in the Osteoporosis group compared to the NC group, whereas Galectin-3 and STING concentrations were significantly reduced. All significant between-group differences are indicated with exact P values directly in **Figure 1** (dot plots showing all individual data points, means, and SD). Notably, all four factors exhibited a graduated alteration pattern across the NC, Osteopenia, and Osteoporosis groups. Comprehensive data are provided in **Table 3**.

**Table 3:** Comparison of the levels of CXCL-2, FYN, Galectin-3, STING and other bone metabolic markers between the three groups .

	NC group	Osteopenia group	Osteoporosis group
Variables	(n=100)	(n=100)	(n=100)
ALP(U/L)	92.48 ± 10.66	96.35 ± 9.30^*^	122.39 ± 33.09^*△^
β-CTX(ng/ml)	0.51 ± 0.14	0.58 ± 0.12^*^	0.67 ± 0.26*^△^
PINP(ng/ml)	53.18 ± 11.93	55.38 ± 15.06	60.77 ± 19.93^*^
CXCL-2(pg/ml)	53.07 ± 13.97	56.13 ± 14.30	62.27 ± 13.53*^△^
FYN(pg/ml)	0.30 ± 0.06	0.38 ± 0.05^*^	0.44 ± 0.07*^△^
Galectin-3(pg/ml)	633.54 ± 185.20	440.88 ± 105.03^*^	326.55 ± 75.65*^△^
STING(pg/ml)	123.86 ± 18.23	117.72 ± 22.80	115.68 ± 18.07^*^

Data were presented as mean ± SD. NC group, normal control subjects. ^*^P<0.05 compared with group NC, ^△^P<0.05 compared with group osteopenia.

β-CTX, Serum type I collagen cross-linked C-terminal peptide β-specific sequence; P1NP, serum type I procollagen N-terminal propeptide; ALP, alkaline phosphatase; CXCL-2, C-X-C motif chemokine ligand 2; STING, stimulator of interferon genes.

Furthermore, the Osteoporosis group displayed markedly elevated concentrations of PINP (P = 0.004), β-CTX (P < 0.001), and ALP (P < 0.001) versus the NC group. The Osteopenia group demonstrated elevated ALP (P = 0.020) and β-CTX (P = 0.001) concentrations versus the NC group. Comprehensive data are provided in **Table 3**.

### 3.4 Pearson correlation analysis between CXCL-2, FYN, Galectin-3, STING and other clinical or laboratory parameters

To assess whether these immune factors are specifically linked to skeletal health, we performed Pearson correlation analysis between each cytokine and clinical parameters (**Table 4**). Correlation strength was interpreted using standard criteria: |r| < 0.3 = weak, 0.3 ≤ |r| < 0.5 = moderate, |r| ≥ 0.5 = strong.

**Table 4:** Correlation of serum CXCL-2, FYN, Galectin-3 and STING with other measured clinical and laboratory parameters in different groups.

	CXCL-2		FYN		Galectin-3		STING	
Variables	r	*P-value*	r	*P-value*	r	*P-value*	r	*P-value*
Age(years)	-0.113	0.051	0.011	0.853	-0.035	0.551	-0.054	0.348
BMI(kg/m^2^)	0.043	0.454	-0.019	0.748	-0.008	0.896	0.065	0.259
ALT(U/L)	0.020	0.735	0.005	0.926	-0.015	0.796	-0.023	0.685
AST(U/L)	0.003	0.960	-0.007	0.904	-0.014	0.803	-0.059	0.309
SCr(umol/L)	0.042	0.471	0.018	0.761	0.045	0.432	-0.144	0.012
TC(mmol/L)	0.075	0.193	-0.019	0.741	0.011	0.847	-0.069	0.232
TG(mmol/L)	-0.073	0.209	-0.028	0.626	-0.018	0.760	0.087	0.132
FBG(mmol/L)	0.010	0.863	-0.007	0.907	-0.014	0.807	0.056	0.330
Ca(mmol/L)	-0.014	0.810	-0.059	0.312	0.026	0.657	-0.056	0.336
P(mmol/L)	-0.068	0.237	-0.076	0.189	0.081	0.161	**0.159**	**0.006**
PTH(pg/ml)	0.075	0.193	0.061	0.289	**-0.152**	**0.009**	-0.077	0.182
25-(OH) D(ng/ml)	**-0.165**	**0.004**	**-0.414**	**<0.001**	**0.497**	**<0.001**	**0.135**	**0.020**
CT(pg/ml)	0.022	0.700	-0.076	0.187	-0.027	0.637	0.036	0.530
β-CTX(ng/ml)	0.098	0.090	**0.208**	**<0.001**	**-0.273**	**<0.001**	-0.056	0.330
PINP(ng/ml)	0	0.994	**0.174**	**0.002**	-0.075	0.194	-0.105	0.070
ALP(U/L)	**0.137**	**0.018**	**0.345**	**<0.001**	**-0.360**	**<0.001**	-0.071	0.218
FN BMD(g/cm^2^)	**-0.281**	**<0.001**	**-0.667**	**<0.001**	**0.662**	**<0.001**	**0.155**	**0.007**
TH BMD(g/cm^2^)	**-0.183**	**0.001**	**-0.656**	**<0.001**	**0.618**	**<0.001**	**0.162**	**0.005**
LS BMD(g/cm^2^)	**-0.230**	**<0.001**	**-0.642**	**<0.001**	**0.602**	**<0.001**	**0.156**	**0.007**
CXCL-2(pg/ml)	1	–	**0.219**	**<0.001**	-0.111	0.054	-0.041	0.478
FYN(pg/ml)	**0.219**	**<0.001**	1	–	**-0.514**	**<0.001**	-0.111	0.055
Galectin-3(pg/ml)	-0.111	0.054	**-0.514**	**<0.001**	1	–	0.110	0.058
STING(pg/ml)	-0.041	0.478	-0.111	0.055	0.110	0.058	1	–

r, pearson correlation coefficient. P-value: correlation was significant at the 0.05 level.

Bold values indicate statistical significance (P < 0.05).

As shown in **Table 4**, CXCL-2 concentrations demonstrated weak positive correlations with ALP (r = 0.137, P = 0.018) and FYN (r = 0.219, P < 0.001), and weak negative correlations with 25-(OH)D (r = -0.165, P = 0.004) and BMD at all sites (r ranging from -0.183 to -0.281, P = 0.001 to <0.001).

FYN concentrations displayed moderate positive correlations with β-CTX (r = 0.208, P < 0.001) and ALP (r = 0.345, P < 0.001), a weak positive correlation with PINP (r = 0.174, P = 0.002), and strong negative correlations with 25-(OH)D (r = -0.414, P < 0.001), BMD at all sites (r ranging from -0.642 to -0.667, all P < 0.001), and Galectin-3 (r = -0.514, P < 0.001).

Galectin-3 showed moderate to strong positive correlations with 25-(OH)D (r = 0.497, P < 0.001) and BMD at all sites (r ranging from 0.602 to 0.662, all P < 0.001), and weak to moderate negative correlations with PTH (r = -0.152, P = 0.009), β-CTX (r = -0.273, P < 0.001), ALP (r = -0.360, P < 0.001), and FYN (r = -0.514, P < 0.001).

STING displayed weak positive correlations with P (r = 0.159, P = 0.006), 25-(OH)D (r = 0.135, P = 0.020), and BMD at all sites (r ranging from 0.155 to 0.162, P = 0.005 to 0.007), and a weak negative correlation with SCr (r = -0.144, P = 0.012).

Importantly, while these molecules demonstrated strong correlations with BMD at all measured sites, they exhibited no significant correlations with a broad panel of non-skeletal systemic parameters including age, BMI, ALT, AST, TC, TG, and FBG (all P > 0.05, ranging from 0.051 to 0.960), with the exception of a significant negative correlation between STING and SCr (P = 0.012). This selective association suggests that their dysregulation is specifically linked to skeletal pathophysiology rather than generalized systemic disturbances.

### 3.5 Regression analysis of factors correlated with CXCL-2, FYN, Galectin-3 and STING levels

To identify independent predictors of each cytokine level while adjusting for potential confounders, multiple linear regression analysis was performed (**Table 5**). Regression coefficients (β) represent the change in cytokine level per unit change in the predictor variable, with 95% confidence intervals indicating the precision of these estimates.

**Table 5:** Results of a multivariate linear model of selected variables performed for CXCL-2, FYN, Galectin-3 and STING levels.

Variables	Independent variable	β	SE	β'	t	*P-value*	95% CI lower	95% CI upper
CXCL-2(pg/ml)	25-(OH) D	0.040	0.176	0.017	0.227	0.820	-0.307	0.387
	ALP	0.017	0.038	0.029	0.445	0.656	-0.058	0.091
	**FN BMD**	**-33.717**	**10.736**	**-0.386**	**-3.141**	**0.002**	**-54.846**	**-12.588**
	**TH BMD**	**24.999**	**11.405**	**0.256**	**2.192**	**0.029**	**2.552**	**47.446**
	LS BMD	-5.405	10.013	-0.061	-0.540	0.590	-25.111	14.301
	FYN	15.129	13.410	0.087	1.128	0.260	-11.263	41.520
FYN(pg/ml)	25-(OH) D	0.001	0.001	0.077	1.379	0.169	0.000	0.003
	ALP	0.000	0.000	-0.012	-0.252	0.801	0.000	0.000
	PTINP	0.000	0.000	0.061	1.392	0.165	0.000	0.001
	β-CTX	-0.022	0.019	-0.051	-1.120	0.263	-0.060	-0.016
	**FN BMD**	**-0.141**	**0.048**	**-0.280**	**-2.943**	**0.004**	**-0.236**	**-0.047**
	**TH BMD**	**-0.130**	**0.049**	**-0.231**	**-2.642**	**0.009**	**-0.227**	**-0.033**
	**LS BMD**	**-0.099**	**0.043**	**-0.193**	**-2.292**	**0.023**	**-0.184**	**-0.014**
	CXCL-2	0.000	0.000	0.060	1.374	0.171	0.000	0.001
	**Galectin-3**	**0.000**	**0.000**	**-0.115**	**-2.015**	**0.045**	**0.000**	**0.000**
Galectin-3(pg/ml)	PTH	-1.115	1.119	-0.044	-0.996	0.320	-3.318	1.088
	25-(OH) D	3.188	1.729	0.106	1.844	0.066	-0.215	6.590
	β-CTX	-39.898	42.793	-0.043	-0.932	0.352	-124.121	44.325
	ALP	-0.173	0.369	-0.023	-0.468	0.640	-0.900	0.554
	**FN BMD**	**376.756**	**104.715**	**0.342**	**3.598**	**<0.001**	**170.660**	**582.852**
	TH BMD	159.147	110.577	0.129	1.439	0.151	-58.485	376.780
	LS BMD	53.308	97.516	0.048	0.547	0.585	-138.619	245.235
	FYN	-234.769	130.197	-0.108	-1.803	0.072	-491.017	21.479
STING(pg/ml)	**SCr**	**-0.401**	**0.153**	**-0.148**	**-2.624**	**0.009**	**-0.702**	**-0.100**
	**P**	**29.043**	**11.416**	**0.145**	**2.544**	**0.011**	**6.575**	**51.511**
	25-(OH) D	0.167	0.248	0.050	0.675	0.501	-0.321	0.656
	FN BMD	-0.558	14.836	-0.005	-0.038	0.970	-29.756	28.641
	TH BMD	11.881	15.711	0.087	0.756	0.450	-19.039	42.801
	LS BMD	4.666	13.944	0.038	0.335	0.738	-22.777	32.108

β, unstandardized coefficients; β', standardized coefficients; SE, standard error; t, t-test statistic; P, correlation was significant at the 0.05 level; min, minimum; max, maximum; 95% CI, confidence interval for the unstandardized regression coefficient (β).

Bold values indicate statistical significance (P < 0.05).

**Figure 2:**
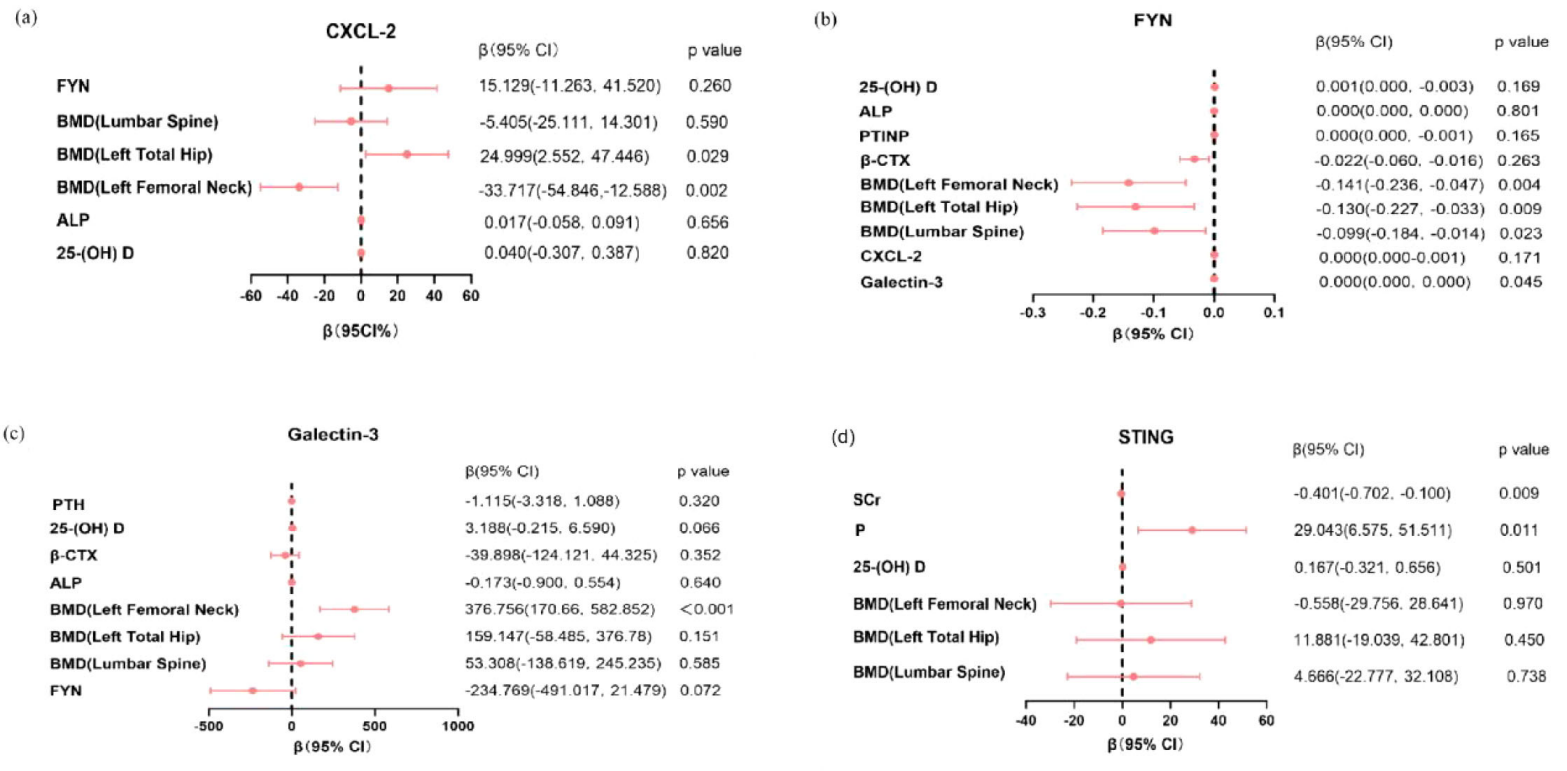
Forest plots showing the regression coefficients and 95% confidence intervals for **(A)** CXCL-2, **(B)** FYN, **(C)** Galectin-3, and **(D)** STING.

As presented in **Table 5** and **Figure 2**, the regression analysis revealed the following significant associations:

CXCL-2 was independently associated with FN BMD (β = -33.717, 95% CI -54.846 to -12.588, P = 0.002) and TH BMD (β = 24.999, 95% CI 2.552 to 47.446, P = 0.029).

FYN was independently associated with FN BMD (β = -0.141, 95% CI -0.236 to -0.047, P = 0.004), TH BMD (β = -0.130, 95% CI -0.227 to -0.033, P = 0.009), LS BMD (β = -0.099, 95% CI -0.184 to -0.014, P = 0.023), and Galectin-3 (β ≈ 0, 95% CI 0.000 to 0.000, P = 0.045).

Galectin-3 was independently associated with FN BMD (β = 376.756, 95% CI 170.660 to 582.852, P < 0.001).

STING was independently associated with SCr (β = -0.401, 95% CI -0.702 to -0.100, P = 0.009) and P (β = 29.043, 95% CI 6.575 to 51.511, P = 0.011).

**Figure 2** presents forest plots visualizing these regression coefficients and their 95% confidence intervals.

### 3.6 ROC analysis of CXCL-2, FYN, Galectin-3 and STING indicating the ability to diagnose osteoporosis

**Figure 3:**
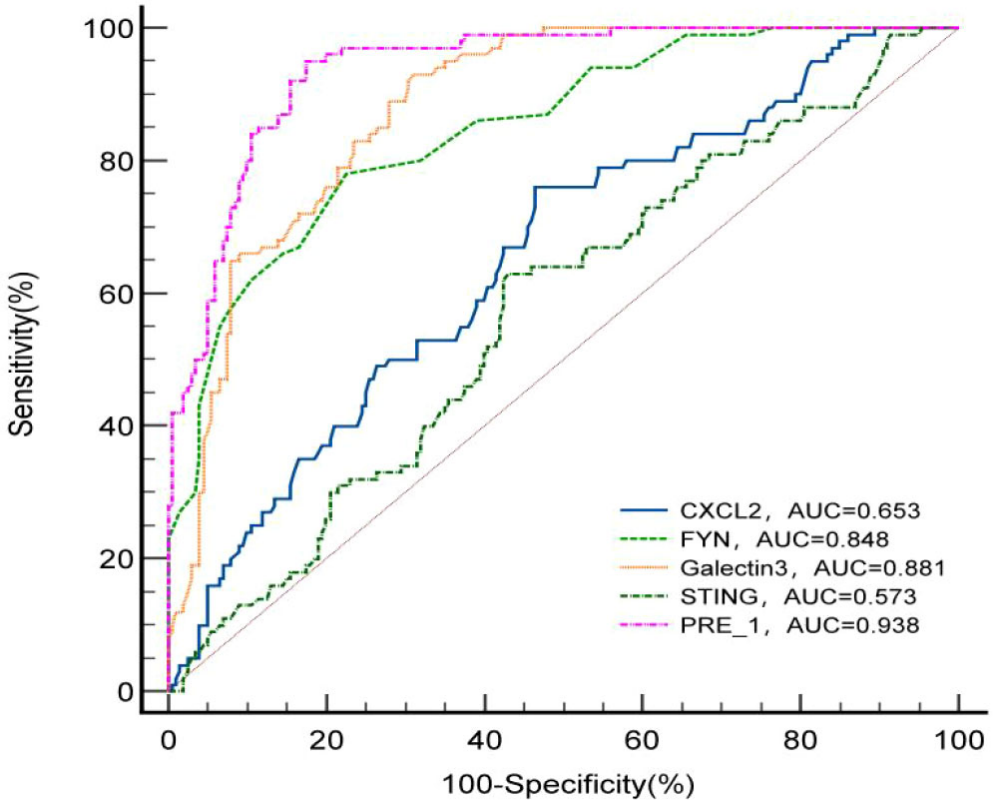
Comparison of receiver operator characteristic curve analysis for prediction of osteoporosis based on CXCL-2, FYN, Galectin-3 and STING levels. PRE_1 was the prediction probability, representing the combined diagnostic efficacy of CXCL-2, FYN, Galectin-3 and STING.

To evaluate the diagnostic utility of each cytokine for PMOP, we performed receiver operating characteristic (ROC) analysis. The area under the curve (AUC) indicates diagnostic accuracy, with values closer to 1.0 representing better discrimination between osteoporosis and non-osteoporosis individuals. An AUC of 0.5 indicates no discriminative ability (random chance). **Figure 3** and **Table 6** display the ROC analysis outcomes for serum concentrations of CXCL-2, FYN, Galectin-3, and STING. The AUC values for STING, FYN, and CXCL-2 were 0.573 (95% CI 0.515-0.630, P = 0.039), 0.848 (95% CI 0.803-0.887, P < 0.001), and 0.653 (95% CI 0.596-0.707, P < 0.001), respectively. Galectin-3 demonstrated an AUC of 0.881 (95% CI 0.839-0.916, P < 0.001), marginally exceeding those of STING, FYN, and CXCL-2. The optimal threshold values for osteoporosis identification were 118.740 pg/mL for STING, 0.390 pg/mL for FYN, and 54.945 pg/mL for CXCL-2. Galectin-3 showed a threshold value of 433.113 pg/mL, achieving 69.0% sensitivity and 93.0% specificity. Additionally, the combined AUC for all four biomarkers reached 0.938, exceeding the AUC of each individual factor. The optimal combined threshold value was 0.250 pg/mL, demonstrating 95.0% sensitivity and 82.5% specificity. Notably, the combined panel outperformed any single marker, supporting the hypothesis that assessing a network of osteoimmune factors may capture the multifaceted dysregulation in PMOP more effectively than individual markers. These results indicate that combining STING, FYN, Galectin-3, and CXCL-2 enhances the prediction performance, suggesting that their combined effect is more significant than their individual contributions in diagnosing PMOP.

**Table 6:** ROC analysis of CXCL-2, FYN, Galectin-3 and STING values for discriminating osteoporosis.

Variable	AUC	95%CI lower	95%CI upper	Cut-off point	Sensitivity	Specificity	*P-value*
CXCL2	0.653	0.596	0.707	54.945	0.760	0.535	0.000
FYN	0.848	0.803	0.887	0.390	0.780	0.825	0.000
Galectin3	0.881	0.839	0.916	433.113	0.690	0.930	0.000
STING	0.573	0.515	0.630	118.740	0.575	0.630	0.039
PRE_1	0.938	0.905	0.963	0.250	0.950	0.825	0.000

The ROC curve was described as a function of 100-specificity(%) for predicting patients with osteoporosis based on the serum concentrations of CXCL-2, FYN, Galectin-3, STING and PRE_1; 95%CI, 95% confidence interval.

PRE_1, prediction probability, representing the combined diagnostic efficacy of CXCL-2, FYN, Galectin-3 and STING.

## 4 Discussion

As the global population ages, PMOP has become a prevalent condition that markedly impacts women’s health, placing a considerable burden on healthcare systems and society at large (Li et al., 2023; Huang et al., 2024). The pathophysiology of PMOP involves estrogen deficiency and the activation of inflammatory factors, which lead to a high-turnover state of bone metabolism. This leads to a notable elevation in bone resorption, along with compensatory bone formation. However, since the rate of resorption exceeds that of formation, net bone loss occurs, ultimately leading to osteoporosis (Li et al., 2023; Huang et al., 2024; Miron et al., 2024). In recent years, osteoimmunology has emerged as a key area of focus in the prevention and treatment of PMOP (Hong et al., 2024). Despite advances in research, the dynamic alterations and functions of immune-associated cytokines (CXCL-2, FYN, Galectin-3, and STING) throughout PMOP development remain inadequately characterized. In this investigation, modifications in serum concentrations of CXCL-2, FYN, Galectin-3, and STING among PMOP patients were examined to evaluate their potential significance for PMOP diagnosis and therapeutic management. The main findings of this research are as follows: (1) Patients with PMOP exhibited elevated levels of FYN and CXCL-2, and decreased levels of Galectin-3 and STING compared to individuals with normal BMD. (2) CXCL-2 concentrations demonstrated positive associations with ALP and FYN but negative associations with 25-(OH)D, BMD at the Left Femoral Neck, Left Total Hip, and Lumbar Spine. FYN concentrations exhibited positive relationships with β-CTX, PINP, ALP, and CXCL-2, while displaying negative relationships with 25-(OH)D, BMD at the Left Femoral Neck, Left Total Hip, Lumbar Spine, and Galectin-3. Galectin-3 concentrations demonstrated positive relationships with 25-(OH)D and BMD at the Left Femoral Neck, Left Total Hip, and Lumbar Spine, but negative relationships with PTH, β-CTX, ALP, and FYN. STING concentrations exhibited positive associations with P, 25-(OH)D, and BMD at the Left Femoral Neck, Left Total Hip, and Lumbar Spine, but negative relationships with SCr. (3) CXCL-2 demonstrated marked associations with BMD at the Left Femoral Neck and Left Total Hip. FYN exhibited marked associations with BMD at the Left Femoral Neck, Left Total Hip, Lumbar Spine, and Galectin-3. Galectin-3 demonstrated marked links to BMD at the Left Femoral Neck. STING exhibited marked associations with SCr and P. (4) Increased concentrations of FYN and CXCL-2, combined with reduced concentrations of Galectin-3 and STING, may contribute to the risk of developing PMOP. Furthermore, Galectin-3 may function as a more dependable biomarker for predicting PMOP. The combined assessment of these four immune-related factors could enhance diagnostic accuracy for PMOP.

Immune cells and bone cells share a common origin in the bone marrow and are spatially adjacent, making it widely recognized that bone tissue and the immune microenvironment exert reciprocal effects on each other under both normal and disease conditions (De Martinis et al., 2020). Over recent years, osteoimmunology has emerged as an interdisciplinary field demonstrating that immune cells and the cytokines they produce are crucial in bone remodeling, particularly in regulating the functions of osteoclasts and osteoblasts (Lorentzon et al., 2022). CXCL-2, a chemokine primarily involved in recruiting inflammatory cells and modulating inflammatory responses (Wang X. et al., 2023), attracts monocytes/macrophages (osteoclast precursors) expressing CXCR2 (C-X-C motif chemokine receptor 2) to the bone surface through chemotaxis, thereby enhancing the local accumulation of these precursor cells. In conditions of estrogen deficiency, CXCL-2 may collaborate with other chemokines, such as CXCL-1 and CXCL-8, to amplify the inflammatory response and create a microenvironment conducive to osteoclastogenesis (Zhai et al., 2023). Jeongim Ha et al. demonstrated that CXCL-2 synergizes with RANKL—an essential mediator of osteoclastogenesis—by enhancing the PI3K/Akt and MAPK/ERK signaling pathways, thus lowering the activation threshold for osteoclast differentiation (Dainichi et al., 2024). In this investigation, CXCL-2 concentrations demonstrated elevation in patients diagnosed with PMOP and exhibited a marked inverse correlation with axial skeleton BMD. These results underscore the pivotal function of inflammation in PMOP pathogenesis and establish a foundation for investigating anti-inflammatory approaches in PMOP therapy. FYN, belonging to the Src family kinases, serves a crucial function in coordinating RANKL and integrin signaling cascades. By phosphorylating adaptor proteins like PYK2 (Proline-rich tyrosine kinase 2), FYN activates small GTPases such as Rac, promoting the assembly of the osteoclast actin ring, which is vital for bone resorption (Kim et al., 2012; Zhao et al., 2022). Additionally, FYN’s interaction with the DAP12/Syk (DNAX activation protein of 12 kDa/Spleen associated tyrosine kinase) signaling axis can enhance RANKL-induced activation of NFATc1 (Nuclear Factor of Activated T-cells cytoplasmic 1), further amplifying osteoclastogenic signaling (Barbu et al., 2010; Tse et al., 2013; Nakamura et al., 2017). In the present study, FYN levels were markedly elevated in patients with PMOP and showed a marked negative correlation with BMD. FYN was also positively correlated with bone resorption markers, such as β-CTX. These results suggest that FYN contributes to excessive bone resorption in PMOP by amplifying the RANKL signaling pathway, a conclusion consistent with previous research highlighting FYN’s critical role in osteoclastogenesis during bone immune regulation. The levels of FYN and CXCL-2 were positively correlated with the bone formation marker ALP, which may indicate a compensatory increase in bone formation driven by the elevated bone turnover rate characteristic of PMOP (Li et al., 2023). Additionally, FYN and CXCL-2 levels showed a negative correlation with 25-(OH)D, likely due to 25-(OH)D deficiency, which leads to increased immune inflammatory factors and enhanced oxidative stress, exacerbating osteoporotic conditions (Athanassiou et al., 2022; Hamza et al., 2023). Based on the findings from this study and prior research, elevated FYN levels in patients with PMOP may promote osteoclast-mediated bone resorption through activation of the RANKL/DAP12/Syk/PYK2/NFATc1 signaling axis. Together, FYN and CXCL-2 are likely to interact through multiple molecular pathways, disrupting bone metabolic homeostasis and contributing to the pathogenesis of PMOP. However, the exact nature of these interactions requires further investigation through targeted functional studies.

Galectin-3, a protein with molecular weight ranging from 29–35 kDa, participates in multiple biological processes including cell adhesion, inflammatory responses, and apoptosis (Simon et al., 2017). Earlier research has demonstrated that Galectin-3 serves a crucial function in bone inflammatory diseases, diabetic complications, and atherosclerosis (Nakajima et al., 2016). Galectin-3 is constitutively expressed in osteocytes, where intracellular Galectin-3 acts as a negative regulator of osteoclastogenesis by promoting apoptosis of osteoclast precursors (Zhang et al., 2024). In bone tissue, Galectin-3 expression undergoes regulation by runt-related transcription factor 2 (Runx2), serving as the principal regulator of osteoblast differentiation and function throughout bone formation processes. *In vitro* investigations have revealed that genetic elimination of Galectin-3, which compromises osteoblast differentiation and activity, correlates with impaired WNT/β-catenin signaling (Iacobini et al., 2018). In this investigation, Galectin-3 concentrations were diminished in patients with PMOP and exhibited a marked positive association with BMD at the left femoral neck. This observation reinforces the protective function of Galectin-3 in bone immune modulation, indicating that decreased concentrations of Galectin-3 may attenuate the suppression of osteoclast formation, thus facilitating osteoporosis progression. Future research should focus on exploring the potential of enhancing bone formation through exogenous supplementation of Galectin-3 or its agonists. STING serves as an essential element of the innate immune signaling cascade, functioning as a pivotal mediator in immune responses triggered by DNA damage and pathogenic infections. Within bone metabolism, STING operates as an innovative modulator of skeletal equilibrium and acts as a primary detector in the innate immune network. After stimulation by cytoplasmic DNA, STING initiates the generation of type I interferons, specifically IFN-β, via the TBK1-IRF3 (TANK-binding kinase 1/Interferon Regulatory Factor 3) signaling pathway. IFN-β is a potent suppressor of osteoclast differentiation, inhibiting RANKL-induced NFATc1 expression. Sustained activation of STING effectively restrains osteoclastogenesis, while loss of STING function accelerates bone resorption and results in increased bone loss (Kwon et al., 2019; Li M. et al., 2024). Additionally, STING serves as an upstream regulator of NF-κB activation and transcription. NF-κB pathway activation suppresses osteogenic differentiation in bone marrow mesenchymal stem cells while enhancing their transdifferentiation toward osteoclasts (Wang Y. et al., 2023). This investigation reveals that STING expression is markedly diminished in patients with PMOP, which may disrupt the equilibrium between bone resorption and formation through impairment of the protective cGAS-STING-TBK1-IRF3-IFN-β signaling pathway. The decline in STING levels may weaken the suppression of osteoclastogenesis, thus exacerbating bone loss. Furthermore, a marked correlation between STING and creatinine levels was observed, potentially resulting from the activation of pro-inflammatory genes by its downstream factor NF-κB, which triggers kidney inflammation. This process could impair glomerular filtration, affecting creatinine and blood phosphorus levels (Han et al., 2022). These findings provide novel perspectives on STING’s function in skeletal immune modulation and indicate its promise as a treatment target for PMOP.

The reviewer’s insightful comment regarding the context-dependent role of STING highlights an important consideration for biomarker development. MacLauchlan et al. demonstrated that STING regulates osteoclastogenesis in a context-dependent manner, with myeloid-specific STING deletion in ovariectomized mice primarily affecting trabecular bone volume (MacLauchlan et al., 2023). This complexity underscores that STING’s utility as a standalone biomarker may be limited. Consistently, our data show that STING alone exhibits modest correlations with BMD (r = 0.15, P < 0.01) and limited diagnostic accuracy (AUC = 0.573). However, when integrated with CXCL-2, FYN, and Galectin-3, the combined panel achieved superior diagnostic performance (AUC = 0.938). While our data demonstrate a strong and selective association between these four factors and BMD, we acknowledge that this does not prove bone-specific immune dysregulation. The circulating nature of these markers and the known systemic effects of menopause on multiple organ systems preclude such a conclusion. Nevertheless, the coordinated dysregulation of these factors may represent an osteoimmune signature particularly relevant to PMOP pathophysiology, warranting further investigation in localized bone marrow studies.

We also recognize that definitive demonstration of pathological significance requires *in vivo* validation in relevant PMOP models, such as ovariectomized mice with cell-specific manipulations. Our clinical findings provide a strong rationale for such investigations, and future studies should focus on dissecting the causal contributions of each factor and validating the therapeutic potential of targeting this network.

## 5 Conclusion

In summary, our study identifies a distinct circulating osteoimmunological signature—comprising elevated CXCL2 and FYN, and reduced Galectin-3 and STING—that is closely associated with disrupted bone-immune homeostasis in PMOP. The dysregulation of this coordinated immune network underscores a critical pathophysiological shift in PMOP, moving beyond a purely metabolic view to one that integrates systemic immune physiology with skeletal integrity. The superior diagnostic performance of this integrated panel, particularly the predictive strength of Galectin-3, validates its utility not merely as a biomarker but as a reflection of the underlying physiological disturbance.

These findings bridge basic osteoimmunology and clinical diagnostics, providing a tangible, physiology-based tool for early PMOP detection. They emphasize the necessity of evaluating skeletal health within the broader context of immune system physiology. Future research should focus on elucidating the precise cellular and signaling pathways through which these molecules govern bone-immune crosstalk, using both *in vitro* and *in vivo* models to establish causality and explore therapeutic potential. Our ongoing mechanistic studies represent an initial step toward this goal.

## Data Availability

The datasets presented in this article are not readily available because they contain clinical information that could compromise patient privacy. Requests to access the datasets should be directed to the corresponding author, Yukun Li (36200702@hebmu.edu.cn), and will be considered subject to institutional ethics committee approval.
